# Biomass carbon accumulation in aging Japanese cedar plantations in Xitou, central Taiwan

**DOI:** 10.1186/1999-3110-54-60

**Published:** 2013-12-03

**Authors:** Chih-Hsin Cheng, Chih-Yu Hung, Chiou-Peng Chen, Chuang-Wun Pei

**Affiliations:** 1 School of Forestry and Resource Conservation, National Taiwan University, Taipei, Taiwan; 2 NTU Experimental Forest, National Taiwan University, Nantou, Taiwan

**Keywords:** Biomass carbon stocks, Ecosystem carbon stocks, Japanese cedar, Mistcherlich model, Stand age

## Abstract

**Background:**

Japanese cedar (*Chrytomeria japonica D. Don*) is an important plantation species in Taiwan and represents 10% of total plantation area. It was first introduced in 1910 and widely planted in the northern and central mountainous areas of Taiwan. However, a change in forest management from exotic species to native species in 1980 had resulted in few new Japanese cedar plantations being established. Most Japanese cedar plantations are now between 30 and 50 years old and reaching their rotation period. It is of interest to know whether these plantations could be viable for future carbon sequestration through the accumulations of stand carbon stocks. Twelve even-aged Japanese cedar stands along a stand age gradient from 37 to 93 years were selected in Xitou of central Taiwan. The study aims were to investigate the basic stand characteristics and biomass carbon stock in current Japanese cedar stands, and determine the relationships among stand characteristics, tree biomass carbon, and stand age.

**Results:**

Our results indicate that existing Japanese cedar plantations are still developing and their live tree biomass carbon continues to accumulate. At stands with a stand age of 90 years, tree density, canopy height, mean diameter at breast height, basal area, and live tree biomass carbon stocks reach to nearly 430 tree ha^-1^, 27 m, 48 cm, 82 m^2^ ha^-1^ and 300 Mg C ha^-1^, respectively.

**Conclusions:**

Therefore, with no harvesting, current Japanese cedar plantations provide a carbon sink by storing carbon in tree biomass.

**Electronic supplementary material:**

The online version of this article (doi:10.1186/1999-3110-54-60) contains supplementary material, which is available to authorized users.

## Background

Carbon (hereafter denoted by “C”) pools and fluxes are receiving increased attention as important factors in global climate change. Forest ecosystems are the primary terrestrial actors in atmospheric CO_2_ uptake and store two-thirds of terrestrial C stocks (Bonan, 2008). Enhancing forest C sequestration as well as the reducing emissions from deforestation and forest degradation has therefore been suggested as an effective means of curbing elevated atmospheric CO_2_ level and mitigating global warming (IPCC 2003, 2006).

The potential for forest C sequestration is a dynamics process and depends on the climatic conditions, species composition, forest age, and management practices (Goodale et al., 2002; Pan et al., 2011). Thus, a well-defined model for estimating forest C sequestration is needed that would enable policymakers to develop comprehensive management programs. However, existing studies focus on temperate forest (Fukuda et al., 2003; Kaipainen et al. 2004). Lack of information in tropical or subtropical forests makes the estimation of forest C inventories and growth dynamics, including those in Taiwan, particularly difficult.

Japanese cedar (*Chrytomeria japonica D. Don*) is one of the most important plantation species in Taiwan, representing 10% of the total plantation area (Forestry Bureau, 1994). It was first introduced in Xitou in 1910. Due to the robust growth performance and the suitable climatic and soil properties (Young, 1975), Japanese cedar was commercially grown over wide areas in the northern and central mountainous areas of Taiwan. The establishment of Japanese cedar plantations reached its peak between 1950 and 1980, but dramatically declined when the focus of national forest management policy shifted from planting exotic species (e.g., Japanese cedar) to native species in 1980. Current stand age for most Japanese cedar plantations in Taiwan ranges between 30 and 50 years and reaches or even exceeds their normal rotation period (40–45 years; Wang 1977). This situation has been effectuated partly by a ban on logging in natural forests, which began in 1990, and partly by unfavorable economics in the cedar industry that led to reductions in harvesting. Because the old plantation forests have generally been thought as a carbon-neutral state (Odum, 1969), it is of interest to quantify if the “aging” processes of the current Japanese cedar plantations could be able for future C accumulation.

Previous studies on Japanese cedar plantations in Taiwan have emphasized biomass C stocks within the rotation period, with the result that little is known on stand characteristics development and biomass C stocks in “old” stands over 50 years of age (Young, 1975; Wang 1977). In this study, twelve even-aged Japanese cedar stands along a stand age gradient from 37 to 93 years were selected. The immediate aims were to (1) investigate the basic stand characteristics of tree density, mean diameter at breast height (DBH), basal area (BA) and canopy height and biomass C stocks in current Japanese cedar stands, and (2) determine the relationships among stand characteristics, tree biomass C, and stand age. The broader aim was to evaluate comprehensively whether Japanese cedar plantations grow beyond the normal rotation age and could be viable for future carbon sequestration through the accumulations of stand carbon stocks. In addition to live tree biomass C stocks, carbon pools in understory vegetation, forest floor, woody debris and soil organic C were also investigated in four of the studied stands.

## Methods

### Study site

The study was conducted in the National Taiwan University Experimental Forest in Xitou, central Taiwan (23°40’N, 120°47’E). The mean annual temperature over the last 30 years is 16.6 °C and the mean annual precipitation is 2,635 mm. Both climatic parameters are shown to be suitable for Japanese cedar (Orwa et al., 2009). The soils, classified as Inceptisols, have developed on the sandstone, siltstone and shale (Chen and Chiang, 1996).

Over the past 100 years, most natural broadleaf forest areas in the Xitou district were replaced by either coniferous or Moso bamboo (*Phyllostachys pubescens*) plantations. Japanese cedar was the most common coniferous species early on, but after the management shift to native species in 1980 this began to be replaced by the Taiwan red cypress (*Chamaecyparis formosensis* Matsum.), China fir (*Cunninghamia lanceolata*), and Taiwania (*Taiwania crytomerioides*). The total area of Japanese cedar plantations in Xitou is 325 ha, comprising 36% of all coniferous plantations by area, and stand age ranged mainly between 40 and 90 years.

The study design consisted of 12 even-aged Japanese cedar stands along a stand age gradient from 37 to 93 years (Table 1). Seven of the selected stands (CJ20, CJ33, CJ41, CJ46, CJ50, CJ62, and CJ73; the numerical values indicate the year of planting) had been thinned, pruned, and weeded under typical forest management practices in Xitou. Five of the selected stands (Xitoufu, Xitoufu 3000, Xitoufu 5400, Xitou 3000, and Kadota; the numerical values indicate the planting density per hectare) were the permanent plots purposely established for the long-term growth monitoring. The stand area in the group of 7 ranged between 2.3 to 17.3 ha, whereas the 5 permanent plots were between 0.1 and 0.15 ha (Table 1). For the 7 selected stands, 3 replicated 20 m × 20 m subplots were established and tree density (# ha^-1^), DBH (cm), BA (m^2^ ha^-1^), and canopy height (m) were measured. Diameter at breast height was measured using calipers and the arithmetic mean was calculated to represent the mean DBH at each survey stand. Canopy height was determined with a laser distance meter (Leica Disto D8, Heerbrugg, Switzerland) and was the mean height of 15–20 dominant trees. The BA for the survey stand was calculated from the sum of BA of each tree inside the plot and divided by plot area. For the permanent plots, tree density, DBH, BA, and canopy height for each tree was recorded every 5–10 years (NTU Experimental Forest, 2010). Both the latest and long-term census data were used in the study.

**Table 1:** **Stand characteristics and live tree biomass carbon stocks of 12 selected even-aged Japanese cedar stands in Xitou, central Taiwan**

	Stand age	Altitude (m)	Stand/plot area (ha)	Density (no ha^-1^)	Mean DBH (cm)	Canopy height (m)	BA (m^2^ha^-1^)	Volume (m^3^ha^-1^)	Tree biomass C (Mg C ha^-1^)	Thinning activities
Typical stands										
CJ73	37	1370	6.3	1358 (162)^a^	23.1 (0.4)	20.8	59.7 (4.0)	520 (34)	165 (10)	-
CJ62	49	1250	3.8	825(95)	28.1(1.3)	24.0	58.1 (1.5)	516 (10)	163 (3)	1990
CJ50	60	1250	16.1	617 (8)	35.3 (1.0)	26.0	63.7 (4.4)	638 (54)	203 (16)	1974, 1992
CJ46	64	1200	7.5	717(17)	36.5 (0.6)	27.0	80.7 (2.9)	828 (34)	262 (10)	1960, 1968
CJ41	69	1150	2.3	658 (36)	33.9 (1.1)	24.1	64.0 (9.2)	678 (68)	203 (20)	1959, 1963
CJ33	77	1250	10.3	685(8)	40.8 (0.5)	30.8	85.3 (2.3)	923 (10)	293 (3)	1959,1962
CJ20	90	1150	3.0	408 (22)	48.6 (1.2)	29.0	77.9 (5.88)	858 (71)	272 (21)	1933, 1959
Permanent plots										
Xitoufu	73	1200	0.15	558	42.2	28.3	81.5	862	274	1928, 1930
Xitoufu 3000	81	1270	0.07	648	40.6	27.3	89.9	953	303	-
Xitoufu 5400	81	1270	0.07	538	39.5	28.1	69.8	729	233	-
Xitou 3000	95	1200	0.13	397	48.8	24.5	78.1	868	277	1928, 1930
Kadota	93	1100	0.15	471	48.5	25.6	90.7	1007	320	1928, 1930

^a^Numbers in parentheses indicate standard error (n = 3).

### Live tree biomass C stocks estimation

The live tree biomass C stocks for each stand was calculated by summing the individual stems within the plot, as illustrated in equation (1).

1 $$\begin{array}{l} \mathrm{Live}\;\mathrm{treebiomassC}\;\left({\mathrm{Mg}\;C\;\mathrm{ha}}^{-1}\right)\\ \;=\left(\mathrm{WV}\times \rho \times \mathrm{EF}\times \mathrm{CF}\right)/A\; \end{array}$$

where WV is stem wood volume (m^3^); ρ is basic wood density (Mg m^-3^); EF is an expansion factor converted from stem biomass to whole tree biomass in the stem, foliage, branches and roots (Mg Mg^-1^); CF is carbon fraction of biomass (%); and A is plot size (ha).

**Figure 1:**
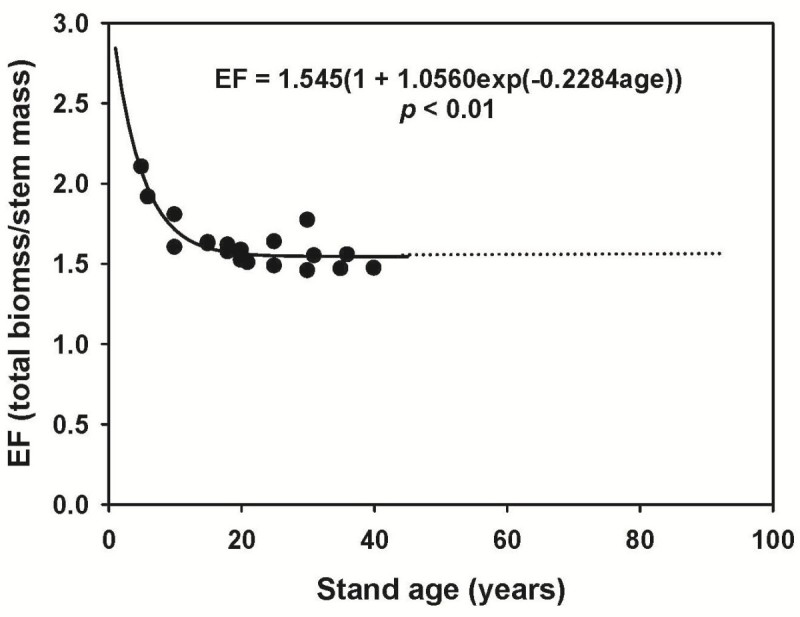
**Expansion factor (EF) as a function of age for Japanese cedar plantations in Xitou, central Taiwan**. Scatter points show the EF of individual stand. The solid line is the regression curve simulated by the Mistcherlich model. An EF value of 1.545 was applied for the Japanese cedar stands with age over 40 y old.

The stem wood volume of individual trees was estimated by using an allometric equation established in the NTU Experimental Forest from 587 trees with ages from 10 to 59 years (Young, 1975).

2 $$H=3.4842\times {\left(\mathrm{DBH}\right)}^{0.5228}+1.3$$

3 $$\begin{array}{ll} \;\log\;V= & -4.193148+0.9333828\\ \times \log\;\left({\mathrm{DBH}}^{2}\times H\right) \end{array}$$

where H is the tree height (m); DBH is the diameter at breast height (cm); and V is the stem volume (m^3^). Stem wood volume was then multiplied by the basic stem density to estimate stem biomass. A recently reported basic wood density value of 0.416 Mg m^-3^ for Japanese cedar in Taiwan was applied (Forest Bureau, 2010). The whole tree biomass in stem, branches, foliage, and roots was estimated by multiplying stem biomass by EF. The EF values for Japanese cedar were calculated from previous studies and are shown in Figure 1. The EFs decreased with increasing age and leveled off at 1.545 when stand age exceeds 30 years. Therefore, a constant EF value of 1.545 was used to calculate the whole tree biomass for all 12 stands in this study. Total biomass was then converted into the carbon weight by multiplying CF, which in this study was taken to be 49.5% (Lin et al., 2002). The obtained EF value was comparable to that for Japanese cedar plantations in Japan (1.54 for stands with stand age older than 40 years; Fukuda et al., 2003), whereas the basic stem density was higher than the average basic density in Japan (0.319 Mg m^-3^; Fukuda et al., 2003). The basic density of Japanese cedar in Taiwan had been reported to vary from 0.302 to 0.442 Mg m^-3^ (Ma et al., 1992; Lin, 2006; Forest Bureau, 2010), while this higher mean basic density in the recent survey is probably due to the use of tree samples with old ages (Kennedy, 1995).

### Carbon pools in understory vegetation, dead organic matter, and soil organic matter

In addition to C pools in living tree biomass, carbon pools in understory vegetation biomass, forest floor, woody debris, and soil organic matter (IPCC, 2006; Peichl and Afrain, 2006) were also estimated in the stands of CJ20, CJ50, CJ58, and CJ73. These C pools were sampled from the three 20 × 20 m subplots as well. The understory vegetation was mainly composed of *Elatostema platyphylloide*, *Angiopteris lygodiifoli* a, *Alocasia macrorrhiza* and *Urtica thunbergiana*. Their C pools were determined by clipping understory biomass at ground level in two randomly-established 2 m × 2 m quadrats. Forest floor was determined by gathering all organic material lying on the forest floor within 0.52 m × 0.52 m quadrat followed by the clipping of understory biomass. Understory biomass and forest floor samples were oven-dried at 65°C to constant mass and weighed. Carbon stocks were calculated by multiplying the dry weight by C fraction. Woody debris (> 2.5 cm in diameter; < 2.5 cm in diameter was considered forest floor) was estimated using the line intersect sampling method. An intersect line was run down the length of a 1 m × 20 m. All woody debris inside this area was collected. Pieces extending outside the area were cut at the boundaries. All debris were oven-dried at 65°C to constant mass and weighed. A small amount was mixed and ground to measure the C fraction. The C pools in woody debris were estimated by multiplying the dry weight by C fraction.

Soil samples were collected using a soil corer 6.37 cm in diameter. Samples were taken at depths of 0–10 cm, 10–20 cm, and 20–30 cm, with sample for each depth range being composited from at least 6 sampling locations within each subplot. Soil samples were air-dried and ground to pass through a 2 mm sieve. A subsample of soils was finely ground using a ball grinder (Oscillating Mill MM400 by Retsch, Newtown, PA, USA) for C analysis. Bulk density was determined using the core method. Stone content was assessed based on field observations based on the menu of the Munsell Soil Color Chart. Soil C stocks were calculated by multiplying C concentration by bulk density, stone contents, and soil horizon thickness.

In this study, the C fraction for understory vegetation biomass, forest floor, woody debris, and soil organic carbon were determined using an elemental analyzer (Perkin Elmer 2400 CHN; Perkin Elmer, Norwalk, CT, USA).

### Age-related stand characteristics development and live tree biomass C accumulation

Live tree biomass C stocks and stand characteristics of tree density, mean DBH, canopy height and basal area are useful indices of stand development (Fain et al., 1994). In this study, the effect of stand age on these values was determined. We first combined the latest census data from the 5 permanent plots with that from 7 selected stands to represent the stand-level growth of Japanese cedar plantations covering with spatial variation. In addition, we assembled data from previous studies (Additional file 1: Table S1) with the current results to model the age-related changes of stand characteristics and live tree biomass C stocks. However, the values of BA and live tree biomass were not provided in some previous studies and simply calculated based on the mean DBH and tree density. Thus, a general age-related trend was focused here rather than the discussion for the interplay among parameters because of the slight differences in the calculations/estimations. Second, the long-term monitoring data from each permanent plot were used to assess trends in stand characteristics development and live tree biomass C stocks accumulation inside each permanent plot.

Stand characteristics and live tree biomass C stocks as functions of stand age were calculated with a common Mitscherlich function (Fukuda et al., 2003) by using SAS version 9.2 as follows:

4 $$\begin{array}{l} \mathrm{Living}\;\mathrm{tree}\;\mathrm{biomass}\;C\;\mathrm{or}\;\mathrm{stand}\;\mathrm{characteristics}\\ \;=m_{1}\times \left(1–m_{2}\times \exp\left(-m_{3}\times \mathrm{stand}\;\mathrm{age}\right)\right) \end{array}$$

where m_1_ is the asymptote, the maximum/minimum number of stand characteristics of tree density, mean DBH, canopy height and basal area or amount of living tree biomass C; and m_2_ and m_3_ are parameters of the Mitscherlich model.

## Results

### Stand characteristics and live tree biomass C stocks

Stand characteristics (tree density, mean DBH, BA, and canopy height) and live tree biomass C stocks for the 12 selected Japanese cedar stands are listed in Table 1. The highest tree density (1,358 trees ha^-1^) was found in CJ73, where no thinning activity had occurred. Stand tree density declined with stand age and reached its lowest point at approximately 430 trees ha^-1^ for the 90-year-old stands. By contrast, the values for stand characteristics, including canopy height, mean DBH, and BA, increased with stand age. Despite decreasing tree density, the increases in DBH and BA caused the living tree biomass to increase. Live tree biomass C stocks for the 12 selected stands ranged from 165 to 320 Mg C ha^-1^ and tended to increase with stand age. For stands at 90 years of age, canopy height, mean DBH, BA, and tree biomass C stocks were nearly 27 m, 48 cm, 82 m^2^ ha^-1^ and 300 Mg C ha^-1^, respectively.

**Figure 2:**
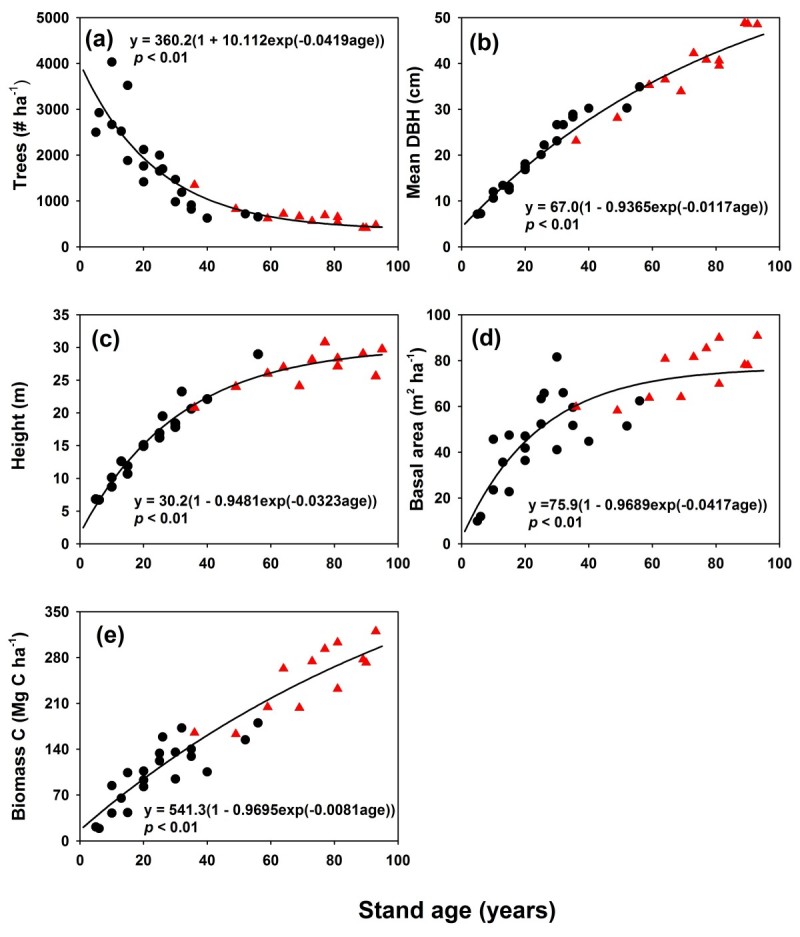
**Age**-**related stand characteristics and live tree biomass C stocks for the Japanese cedar plantations in Xitou**, **central Taiwan. Each point represents a single stand**. The solid line is the regression curve simulated by the Misterchlich model. Black circles are data from previous studies. Red triangles stand for the 12 selected stands in this study. **(a)** tree density, **(b)** mean DBH, **(c)** canopy height, **(d)** basal area, and **(e)** live tree biomass C stocks.

Using data from previous studies, general age-related patterns in tree density, mean DBH, BA, and live tree biomass C stocks were determined (Figure 2). Stand age appeared to have a direct effect on stand characteristics and live tree biomass C stocks. Stand tree density, canopy height and BA took 60 years to reach 90% minimum/maximum values, whereas mean DBH and live tree biomass C stocks remained below the maximum values at the stand age of 90 years. These relationships between stand characteristics and tree biomass stocks and stand age were well regressed by the Mistcherlich model, which yielded potential values of 360 trees ha^-1^, 67 cm, 30 m, 77 m^2^ ha^-1^, and 541 Mg C ha^-1^ for tree density, mean DBH, canopy height, BA, and live tree biomass C stocks, respectively (Figure 2).

**Figure 3:**
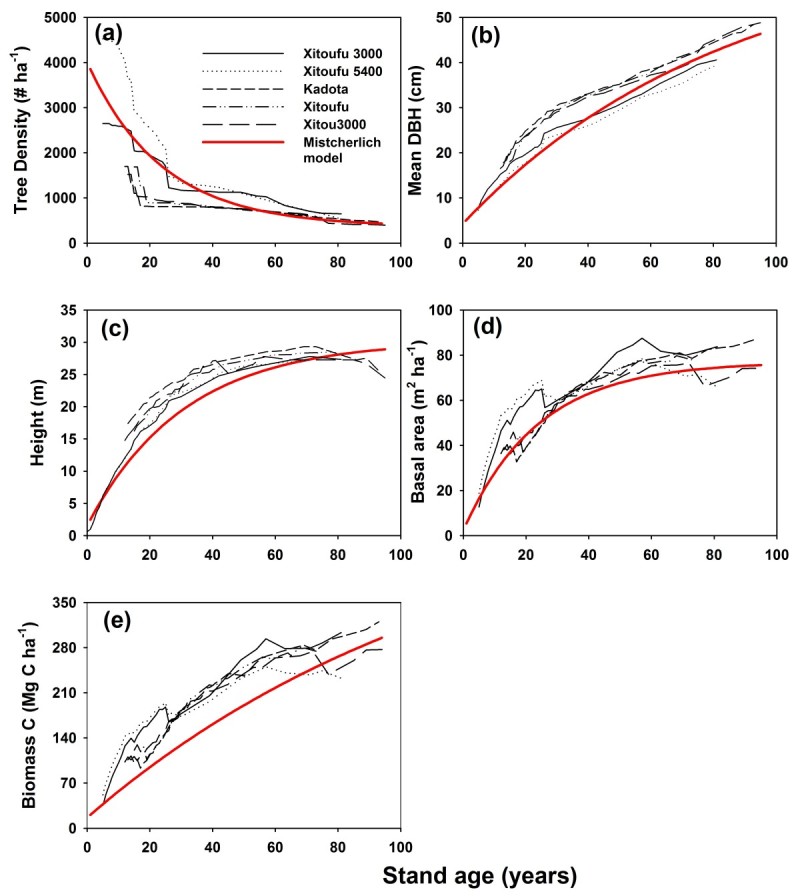
**Age**-**related stand characteristic and live tree biomass C stocks for Japanese cedar plantations in five permanent plots in Xitou**, **central Taiwan**. Each black line represents an individual permanent plot. The red line is the Mistcherlich curve simulated from the spatial data set. **(a)** tree density, **(b)** mean DBH, **(c)** canopy height, **(d)** basal area, and **(e)** live tree biomass C stocks.

The temporal trends in stand characteristics and live tree biomass C stocks from the permanent plots were similar to the trends observed in the spatial data set, in which tree density decreased with stand age, whereas mean DBH, canopy height, BA, and live tree biomass C stocks increased (Figure 3). Thus, both spatial and spectral data sets suggest that live tree biomass C stocks can continue to accumulate in Japanese cedar stands over 90 years old.

### Carbon pools in understory vegetation, dead organic matter and soil organic matter

**Figure 4:**
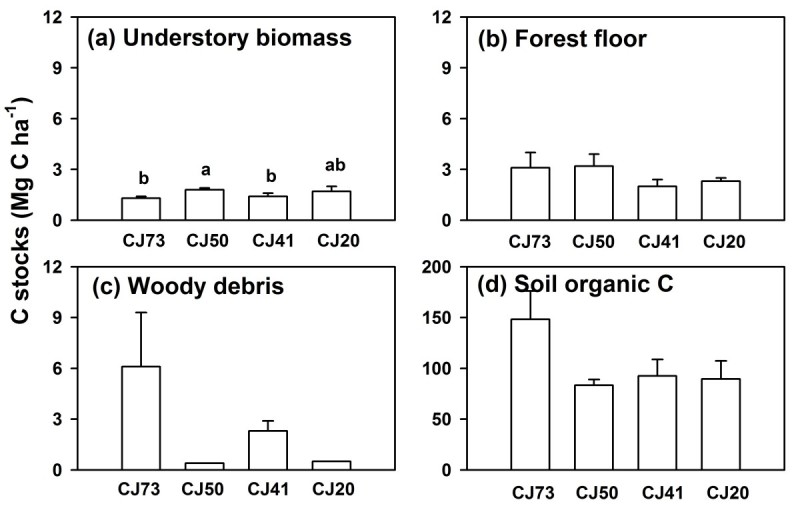
**Carbon stocks in different pools for four Japanese cedar stands with stand ages between 40 and 90 years (a) understory vegetation, (b) forest floor, (c) woody debris, and (d) soil organic matter.** Error bars are standard error of the mean. The different lowercase letters indicate significant differences between stands.

Carbon pools in wood debris, forest floor, and understory vegetation for 4 selected stands ranged from 0.28 to 6.12, from 1.95 to 3.21, and from 1.6 to 2.8 Mg C ha^-1^, respectively (Figure 4). Compared to tree biomass C stocks, these carbon pools were proportionally small (< 5%). In addition, these C pools varied among stands and showed no apparent age-related trends (Figure 4). The C pools in the soil organic matter to a depth of 30 cm ranged between 83 and 148 Mg C ha^-1^, and were comparable with C stocks in the live tree biomass. The C pools in the soil organic matter and tree biomass together comprised more than 95% of ecosystem C stocks. Again, no clear relationship was found between stand age and soil organic C at the 0–30 cm depth range, or between stand age and soil organic C in individual horizons of 0–10, 10–20, and 20–30 cm (data not shown).

## Discussion

### Relationship of C stocks to stand age

Our results derived from both spatial and temporal data sets indicate that the stand characteristics of tree density, mean DBH, canopy height and basal area for present Japanese cedar plantations in Xitou are still developing and that live tree biomass C stocks are also continually accumulating. These results are in agreement with the findings of studies of Japanese cedar plantations in Japan that found biomass C continued to accumulate after 90 years of age (Fukuda et al., 2003; Takeuchi, 2005; Sasaki and Kim, 2009). Many studies from other regions and on other species have similarly shown living tree biomass C stocks accumulating in stand scale that are either over 90 years old (Law et al., 2003; Pregitzer and Euskirchen, 2004) or behind their normal rotation period (Kaipainen et al. 2004; Foley et al., 2009). In contrast, some studies indicate that live tree biomass C stocks may begin leveling off at 90 years (Shoch et al., 2009; Bradford and Kastendick, 2010). Our results disprove previous ideas that some silviculturiests assumed the increments of Japanese cedar stands becoming “stagnated” behind 60 years in Taiwan (Liu et al., 1977; Liu and Horng, 1978), wherein the old stands with age over 60 years were not included in those studies. In addition, the results of the increase in DBH with stand age provide the direct effect on increasing live tree biomass C stocks with stand age, even though the tree density, canopy height and BA are leveled off after stand age behind 60 years. These linkages between stand characteristics of tree density, mean DBH, canopy height and basal area and living tree biomass C truly improve our knowledge of the prior studies.

Live tree biomass C stocks of old-growth Japanese cedar stands in this study fell within the range of C stocks values for old-growth stands in Japan, which have been variously estimated at 131–322 Mg C ha^-1^ in 77 - 126-year-old stands (converted at root/total biomass ratio at 0.20 and carbon fraction at 0.5; Enoki et al., 2010); 234–459 Mg C ha^-1^ in 94-year-old stand (Masaki et al., 2006), and 219–415 Mg C ha^-1^ in 101- 200-year-old stands (converted at basic stem density at 0.319, carbon fraction at 0.5, and EF at 1.54; Takeuchi, 2005). Fukuda et al. (2003) estimated live tree biomass C stocks for Japanese cedar plantations across Japan and proposed a range of 162–230 Mg C ha^-1^ at 90 year of age, which is less than our estimation. The difference between our estimation and that of Fukuda et al. (2003) may be due to the different basic wood density values used: 0.416 Mg m^-3^ in this study compared to 0.319 Mg m^-3^ in Japan. The higher tree biomass C in Taiwan is in part because the trees are thinned less than there in Japan (Chiba, 1998). Chiba (1998) suggested that reducing thinning may help maintain stand biomass C density.

Although biomass C stocks have the highest C density in older stands, the annual biomass C increment calculated from the Mistcherlich model (Figure 2(e)) decreases with stand age from 3.35 Mg C ha^-1^ year^-1^ at 30 years of age to 2.65 and 2.06 Mg C ha^-1^ year^-1^ at 60 and 90 years, respectively. This decline in annual biomass C accumulation in older stands is similar to that reported in other studies (Binkley et al., 2002; Ryan et al., 2004), implying that younger stands have a larger C sink capacity. The mean value of annual biomass C increments for Japanese cedar plantations in Xitou is higher than the mean values for Japanese cedar plantations in Japan (2.1 Mg C ha^-1^; Fukuda et al., 2003), but less than those from several specific experimental plots, including a 40-year-old plot in Takayama (4.1 Mg C ha^-1^ yr^-1^; Yashiro et al., 2010) and a 31-year-old and a 71 year-old plot in Tottori (7.0 and 5.4 Mg C ha^-1^ yr^-1^; Hosoda, 1999).

In addition to the relationship between tree biomass C stocks and stand age, changes in stand characteristics of tree density, mean DBH, canopy height and BA, were also manifest. Compared to young stands, old Japanese cedar stands feature the following characteristics: (1) lower tree densities, (2) larger stem DBH distributions across size classes, and (3) higher BA values and greater tree height, yet these two features become asymptotic when stand age exceed 60 years. Some other stand characteristics often found in natural old-growth forests, for example, understory reinitiation and coarse woody debris accumulation (Tyrrell and Crow, 1994), are not present in the older Japanese cedar plantations in Xitou, perhaps because of thinning, weeding or residual log removal that are practiced.

Carbon pools in woody debris, understory vegetation, forest floor, and soil organic matter showed no clear age-related pattern in this study. This is perhaps also because of the stand management operations undertaken in Xitou. In addition, some studies have suggested that over time these components may stabilize at steady-state level (Shutou and Nakane, 2004; Jandl et al., 2007; Nave et al., 2010). For instance, Shutou and Nakane (2004) indicate that C pools in soil organic matter and forest floor study in Japanese cedar stands level off 40 years after clear-cutting, which is in agreement with our findings that stand aged between 37 and 90 years showed no significant difference with stand age (Figure 4). Because C pools in understory vegetation, dead organic matter, and soil organic matter do not change in late stages of stand development, most C sequestration occurs in the live tree biomass C. However, further long-term temporal observation is needed to evaluate the changes of C stocks in these pools accurately.

### Implications

In this study, the age-related model is established based on the spatial and spectral data sets and covered with a wide range of stand age (6–93 years) and stand number (33 stands in the 325 ha area). We believe that such sampling strategy is adequate to avoid the particular influences from an individual site and provide relevant information to understand the dynamics of stand characteristics and live tree biomass C stocks for the Japanese cedar plantations in Xitou, central Taiwan. The age-related stand development patterns found in the present study highlight the potential of existing Japanese cedar plantations for future C accumulation. Moreover, the research model used here can be expanded to estimate nationwide biomass C stocks and near-term C accumulation rates. Live tree biomass C stocks of Japanese cedar plantations in other areas of Taiwan are shown in Additional file 1: Figure S1. The similarity between the overall trend in those studies and that the trend in Xitou encourages a wider application of the present model. Based on the data from Forestry Bureau (1994), total area of Japanese cedar plantations is around 40,000 ha and its average live tree biomass C stocks that are calculated by the present model is estimated to be 182 Mg C ha^-1^. This figure is expected to increase to 209 Mg C ha^-1^ by the year of 2022, with an annual biomass C accumulation of 2.7 Mg C ha^-1^ y^-1^ (Table 2). Net amounts of C sequestration in tree biomass for whole Japanese cedar plantations will be 1.09 × 10^6^ Mg C during the period 2012–2022, equal to an annual increase of 1.09 × 10^5^ Mg C y^-1^. With no harvesting, Japanese cedar plantations in Taiwan provide a C sink by storing carbon in tree biomass. The value of annual C accumulation in Japanese cedar plantation equals to 13% of the annual fossil fuel consumption from agriculture sector (8.59 × 10^5^ Mg C y^-1^; Environmental Protection Administration, 2012), but only 0.2% compared to the whole fossil fuel consumption in Taiwan (6.84 × 10^7^ Mg C y^-1^, Environmental Protection Administration, 2012).

**Table 2:** **Total area and total calculated live tree biomass C stocks and the increments of C stocks in 2012–2022 period for Japanese cedar plantations in Taiwan**

Stand age (years)	Total area (ha)	Total biomass C stocks^a^(x 10^3^Mg)	∆ C in 2022 (x 10^3^Mg)
>80	781	215	15
70-80	3,606	906	78
60-70	1,524	349	36
50-60	8,201	1,657	209
40-50	13,150	2,245	369
30-40	12,710	1,882	380
20-30	194	24	6
< 20	0	0	0
Total	40,169	7,287	1,093
Area-weighted C stocks		182	209
(Mg C ha^-1^)		(in 2012)	(in 2022)

^a^Calculated equation: [541.3×(1–0.9695×exp (-0.0081× age))] × area.

In addition to the increases in biomass C stocks, the average tree DBH also increases with age (Figure 2b). The increase in tree DBH has been proposed as a mechanism for C sequestration because a greater C portion can be allocated into long-lived wood products generated from harvested timber (Kaipainen et al., 2004; Foley et al., 2009). Therefore, ageing process of Japanese cedar plantations may then be a legitimate forest management activity for the reduction of greenhouse gas emissions (IPCC, 2003; Kaipainen et al., 2004; Foley et al., 2009). However, information for quantitative estimation has not been established in Taiwan and further research must be conducted.

Although this study proposes Japanese cedar plantations as sites for C sequestration, the domestic and industrial demand for timber cannot be ignored. Otherwise, the reduced harvest would have an effect on shifting wood harvest elsewhere or shifting to other products that require more carbon to produce (Gustavsson and Sathre, 2006). The data on stand characteristics development and biomass C accumulation of Japanese cedar plantations presented here should, therefore, be incorporated with similar data for other species to create a comprehensive database for forestry management in Taiwan.

## Conclusion

Our results indicate that present Japanese cedar plantations in the Xitou area of Taiwan are still developing and that live tree biomass C stocks continue to accumulate beyond the normal rotation period or even beyond a stand age of 90 years. If Japanese cedar stands are not harvested, they can provide a C sink by storing carbon in tree biomass. In association with the increases in tree DBH with stand age, maintaining this ageing process can be a forest management mechanism for the reduction of greenhouse gas emissions.

## Electronic supplementary material

Additional file 1: Table S1: Previous studies of stand characteristics and live tree biomass C stocks of even**-** aged Japanese cedar stands in Xitou, central Taiwan. **Figure S1.** Japanese cedar live tree biomass C stocks in Taiwan. (DOC 58 KB)

Below are the links to the authors’ original submitted files for images.

Authors’ original file for figure 1

Authors’ original file for figure 2

Authors’ original file for figure 3

Authors’ original file for figure 4
