# Supplementary material for: Biomass carbon accumulation in aging Japanese cedar plantations in Xitou, central Taiwan
Source: Bot Stud. 2013 Dec 3;54:60. doi: 10.1186/1999-3110-54-60 (PMC5432850; doi:10.1186/1999-3110-54-60)
Supplement: Supplementary file 1 — Additional file 1: Table S1: Previous studies of stand characteristics and live tree biomass C stocks of even- aged Japanese cedar stands in Xitou, central Taiwan. Figure S1. Japanese cedar live tree biomass C stocks in Taiwan. (DOC 58 KB) [file 40529_2012_52_MOESM1_ESM.doc]

**Additional file 1**

**Table S1.** Previous studies of stand characteristics and live tree biomass C stocks of even-aged Japanese cedar stands in Xitou, central Taiwan.

| References | Age | Density | Mean DBH | Height | BA | Biomass C stocks |
| --- | --- | --- | --- | --- | --- | --- |
|  | (years) | (# ha^-1^) | (cm) | (m) | (m^2^ ha^-1^) | (Mg C ha^-1^) |
| Tai, 1964 | 13 | 2523 | 13.4 | 12.6 | 35.6 | 70 |
| Young, 1972 | 20 | 1418 | 18.1 | - | 36.3 | 89 |
| NTU Experimental Forest, 1974 | 32 | 1187 | 26.6 | 23.2 | 65.9 | 186 |
|  | 56 | 652 | 34.9 | 29.0 | 62.3 | 195 |
| Wang, 1977; Lee, 1978 | 5 | 2496 | 7.1 | 6.8 | 9.9 | 21 |
|  | 10 | 4030 | 12.0 | 10.1 | 45.6 | 84 |
|  | 15 | 3520 | 13.1 | 11.9 | 47.4 | 103 |
|  | 20 | 2120 | 16.8 | 15.1 | 47.0 | 106 |
|  | 25 | 1997 | 20.1 | 16.2 | 63.3 | 133 |
|  | 30 | 1468 | 26.6 | 18.4 | 81.5 | 135 |
| Yu, 1981 | 6 | 2925 | 7.2 | 6.7 | 11.9 | 19 |
|  | 10 | 2665 | 20.1 | 8.7 | 23.5 | 42 |
|  | 15 | 1880 | 12.4 | 10.7 | 22.7 | 43 |
|  | 20 | 1870 | 17.4 | 14.9 | 41.8 | 93 |
|  | 25 | 1650 | 20.1 | 16.9 | 52.3 | 122 |
|  | 30 | 980 | 23.1 | 17.8 | 41.1 | 94 |
|  | 35 | 820 | 28.3 | 20.6 | 51.6 | 128 |
|  | 40 | 625 | 30.2 | 22.1 | 44.7 | 105 |
| Lu, 1983 | 52 | 716 | 30.5 | - | 51.4 | 167 |
| Feng, 1990 | 26 | 1697 | 22.2 | - | 65.7 | 102 |
|  | 35 | 908 | 28.9 | - | 59.5 | 90 |

**Figure S1.** Japanese cedar live tree biomass C stocks in Taiwan. Solid line is the regression curve for Xitou and scatter points represent other Japanese cedar stands in Taiwan (Liu, 1952; Hung, 1965; Hung, 1966; Hung and Chow, 1967; Liu, 1972; NTU Experimental Forest, 1974; Liu et al., 1977; Liu and Horng, 1978; Chang, 1986; Duh et al., 2011).

**References**

Chang CT (1986) Biomass productivity of Cryptomeria japonica stand in central and northern Taiwan. Master Thesis. Nation Taiwan University, Taipei, Taiwan (in Chinese)

Duh CT, Chiou CM, Lin KC (2011) Estimate of above- and below-ground biomass of a Cryptomeria japonica plantation in Renluen area of Taiwan. Quarterly J Chinese Forestry 44:401–412 (in Chinese)

Feng FL (1990) Studies on the quantitative theory of the stand structure and growth in plantations. PhD Thesis. Nation Taiwan University, Taipei, Taiwan (in Chinese)

Hung LP (1965) Study on the stand growth of being affected by different degrees of thinning for Japanese Cryptomeria plantation (Cryptomeria japonica D. Don) (I). Taiwan Forest Res. Insti. Report. No. 115 (in Chinese)

Hung LP (1966) Study on the stand growth of being affected by different degrees of thinning for Japanese Cryptomeria plantation (Cryptomeria japonica D. Don) (II). Taiwan Forest Res. Insti. Report. No. 130 (in Chinese)

Hung LP, Chow SJ (1967) Studies on the growth of test-tree and of its stands established by different seedling from seeds and cuttings of Cryptomeria japonica. Taiwan Forest Res. Insti. Report. No. 147 (in Chinese)

Lee HH (1978) Study on the growth and tree biomass production in Cryptomeria stands of different age classes. Master Thesis. National Taiwan University, Taipei, Taiwan (in Chinese)

Liu SH (1952) Studies on the growth of some important coniferous trees in A-Li-Shan. Taiwan Forest Res. Insti. Report. No. 34 (in Chinese)

Liu SH (1972) A study on the effects of slopes of site to the stand-growth of Cryptomeria plantations. Quar J Chinese For 6:1–15 (in Chinese)

Liu SC, Horng FW (1978b) Growth and yield of Cryptomeria plantation in Taiwan (II)- A-Li-San region. Taiwan Forestry Res. Insti. Bulletin No. 309 (in Chinese)

Liu SC, Horng FW, Chang LP (1977b) Growth and yield of Crypotmeria Plantaion in Taiwan (I)- Wu-Lai and Wen-San working circles. Taiwan Forestry Res. Insti. Bulletin No. 309 (in Chinese)

Lu HC (1983) Growth model for thinned Cryptomeria plantations. Master Thesis. Nation Taiwan University, Taipei, Taiwan (in Chinese)

NTU Experimental Forest (1974) Growth records of important species in the experimental forests. National Taiwan University Experimental Forest, Nantou, Taiwan, p 473 (in Chinese)

Tai KY (1964) Precommercial thinning in Cryptomeria plantation. Taiwan For Science J 1:96–107 (in Chinese)

Wang ZT (1977b) Tree biomass production in Cryptomeria stands of different age classes. J Agri Asso China 102:59–76

Young YC (1972) Effects of thinning on the growth of seeding and grafting. NTU Exp Forest 98:1–18 (in Chinese)

Yu HM (1981) Aboveground biomass and net production in Cryptomeria stands of different age classes. Master Thesis. Nation Taiwan University, Taipei, Taiwan (in Chinese)
