# Supplementary figures and images for: Biomass carbon accumulation in aging Japanese cedar plantations in Xitou, central Taiwan
Source: Bot Stud. 2013 Dec 3;54:60. doi: 10.1186/1999-3110-54-60 (PMC5432850; doi:10.1186/1999-3110-54-60)

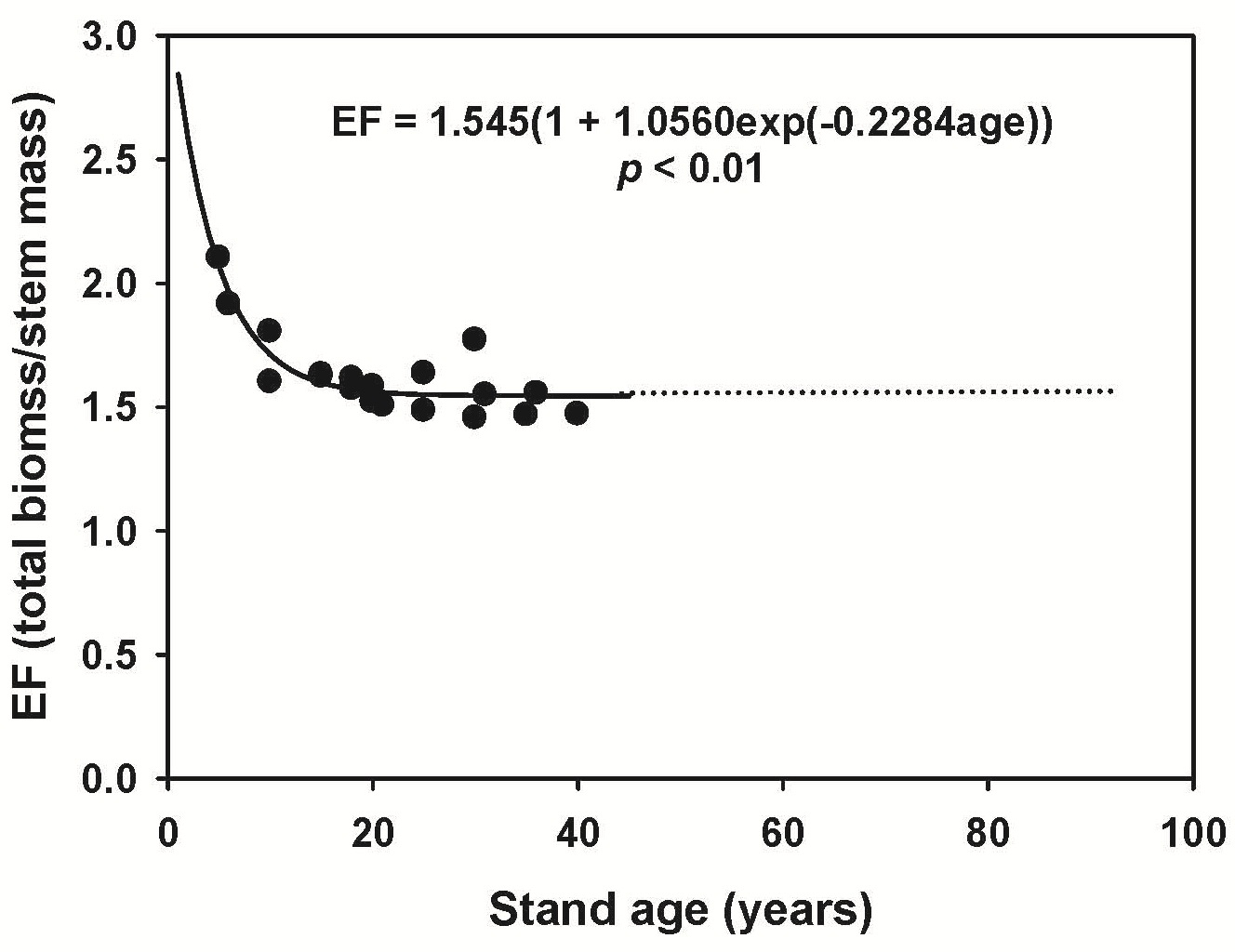

Supplement: Supplementary file 2 — Authors’ original file for figure 1 [file 40529_2012_52_MOESM2_ESM.jpeg]

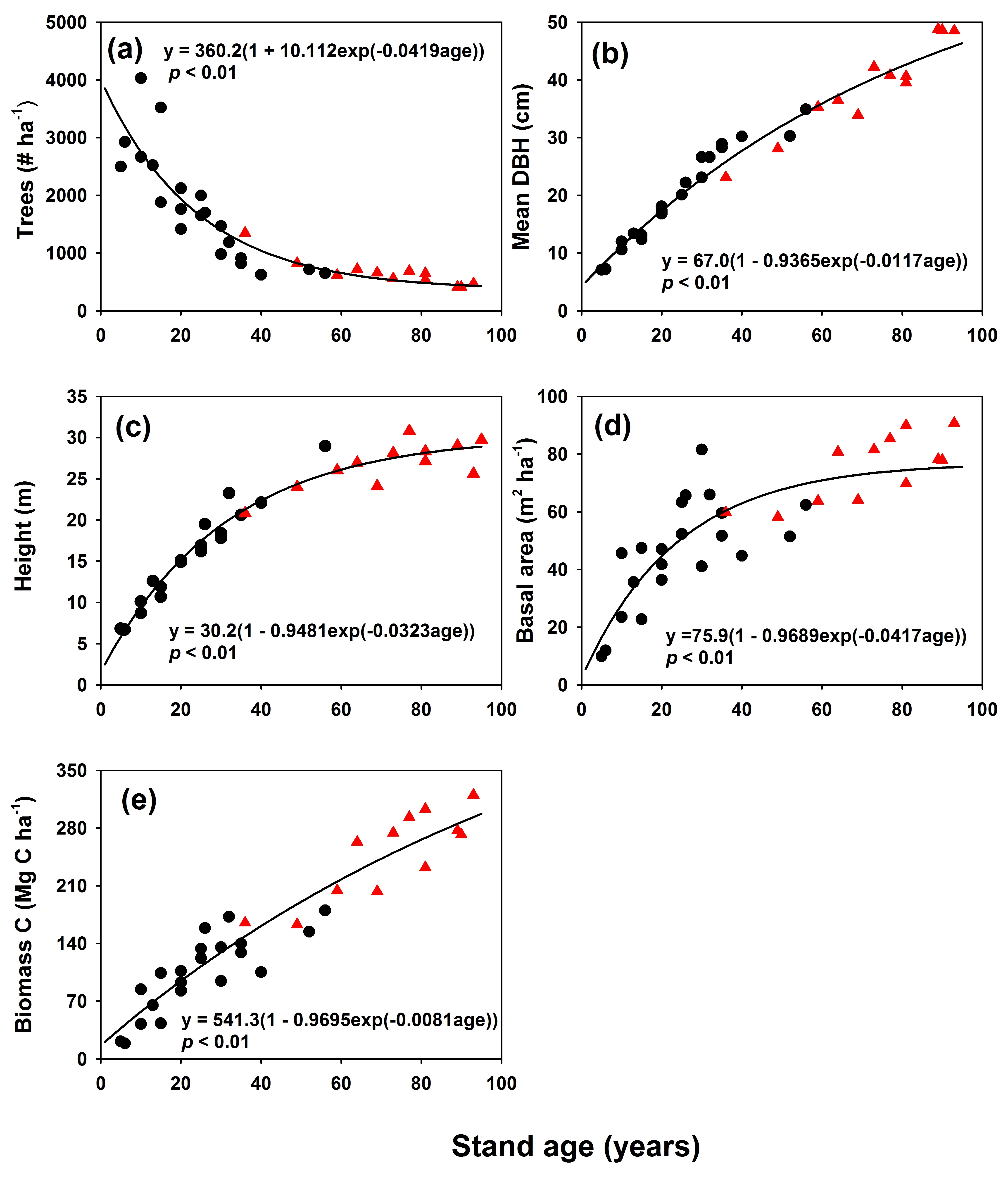

Supplement: Supplementary file 3 — Authors’ original file for figure 2 [file 40529_2012_52_MOESM3_ESM.jpeg]

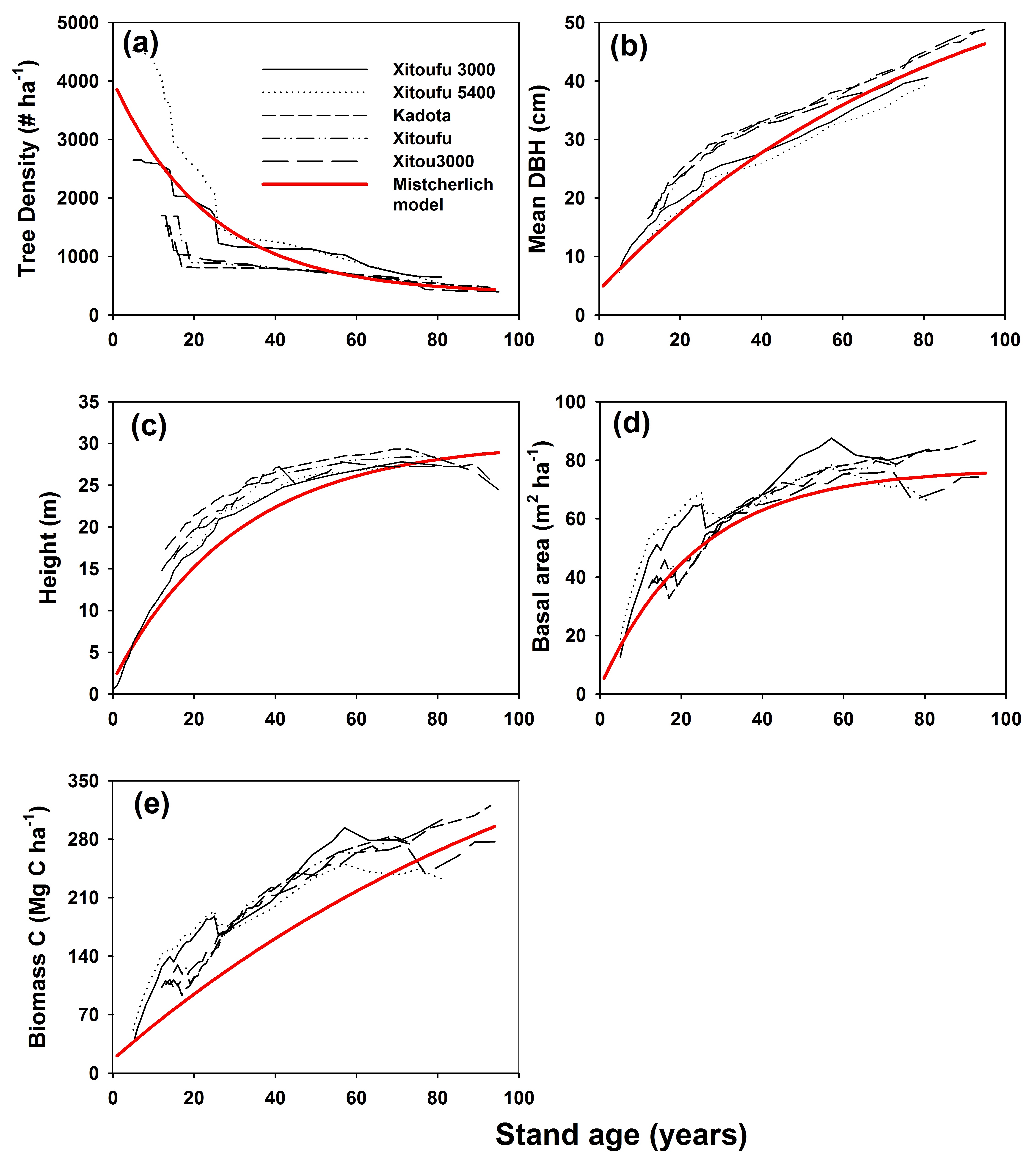

Supplement: Supplementary file 4 — Authors’ original file for figure 3 [file 40529_2012_52_MOESM4_ESM.jpeg]

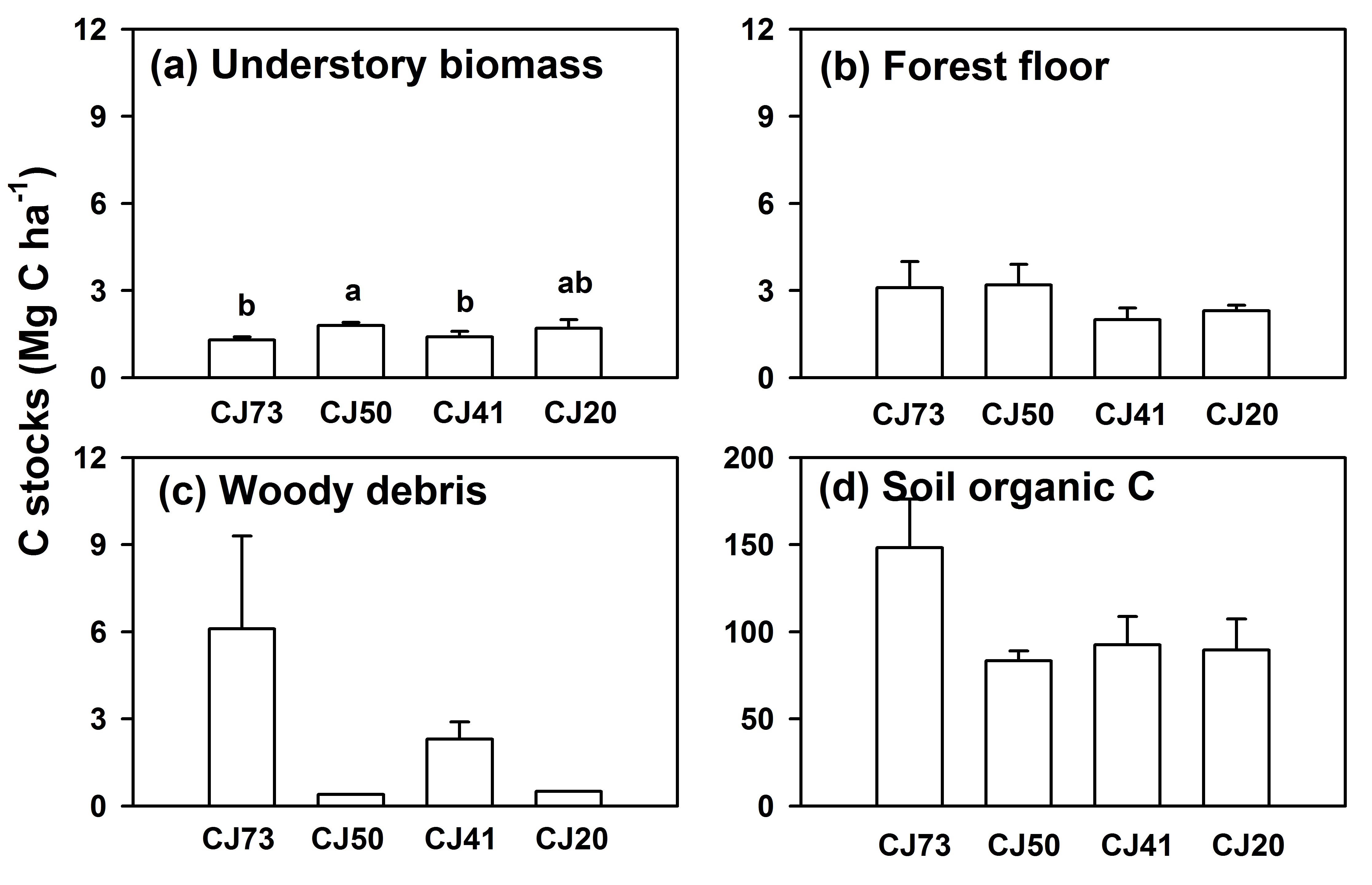

Supplement: Supplementary file 5 — Authors’ original file for figure 4 [file 40529_2012_52_MOESM5_ESM.jpeg]
